# Zinc Deficiency-induced Hypogeusia in a Patient with Refractory Iron-deficiency Anemia: A Case Report

**DOI:** 10.7759/cureus.6365

**Published:** 2019-12-12

**Authors:** Alex J Gooding, Clifford D Packer, Amanda L Pensiero

**Affiliations:** 1 Internal Medicine, Case Western Reserve University School of Medicine, Cleveland, USA

**Keywords:** iron-deficiency anemia, hypogeusia, taste disturbances, malnutrition, oral zinc supplementation, zinc deficiency

## Abstract

Zinc deficiency is a relatively common condition in various American populations but is frequently unrecognized and under-diagnosed. It typically presents with nonspecific symptoms, including lethargy, immune dysfunction, dermatitis, diarrhea, and decreased taste sensation (hypogeusia). Zinc deficiency often occurs concurrently with iron deficiency and has been linked to more severe clinical manifestations of iron deficiency anemia. We describe a 66-year-old man who presented with weakness and fatigue and was found to have persistent iron-deficiency anemia attributable at least in part to malnutrition caused by zinc deficiency-induced hypogeusia. The hypogeusia rapidly improved and nutritional intake normalized with zinc supplementation.

## Introduction

Zinc is a biologically essential and ubiquitous trace mineral found in a wide array of food such as shellfish, red meat, seeds, and fortified grains [1]. Despite its abundance, the prevalence of inadequate zinc intake worldwide has been estimated at 17%, with 35% to 45% of American adults over the age of 60 not meeting the recommended daily zinc requirement [2-3]. Clinical manifestations of zinc deficiency are multifold and nonspecific but often include disturbances in taste perception. We present the case of a zinc-deficient man found to be objectively undernourished in the setting of a longstanding state of hypogeusia that abruptly improved upon zinc supplementation.

## Case presentation

A 66-year-old African American man presented with a two-week history of worsening fatigue and generalized weakness and was found to have severe microcytic anemia with hemoglobin 5.3 g/dL and mean corpuscular volume 57.8 um. Iron deficiency anemia (IDA) was confirmed with a ferritin of 2 ng/ml (25-250 ng/ml). His medical history was significant for IDA of unknown origin, hepatitis C previously treated with ledipasvir/sofosbuvir, right frontal lobe stroke, GOLD II chronic obstructive pulmonary disease (COPD), hypertension, and hyperlipidemia. He denied abdominal pain, melena, hematochezia, dysphagia, odynophagia, hematuria, hemoptysis, or abnormal bruising. He described a four-to-five-year history of persistent hypogeusia for all foods, which made it difficult for him to eat even when he felt hungry. Of note, he had been admitted two years before for similar symptoms, at which time IDA was first diagnosed. He underwent colonoscopy and esophagogastroduodenoscopy at that time, which revealed only internal hemorrhoids and mild gastritis. At that time, he was prescribed an oral iron supplement but adherence was inconsistent due to mild gastrointestinal symptoms. Small-bowel follow-through, push enteroscopy, nor capsule endoscopy was performed throughout the course of his presentation.

On physical exam, the patient was cachectic with temporal and supraclavicular wasting; the body mass index (BMI) was 18.4. Vital signs were normal. There was no cervical, axillary, or inguinal lymphadenopathy. There were diffuse expiratory wheezes and a 2/6 systolic ejection murmur at the upper left sternal border; the abdomen was soft and nontender, with no masses and no enlargement of the liver or spleen. There was no jaundice and no rashes or other skin lesions and no peripheral edema. The neurologic exam was non-focal.

**Figure 1:**
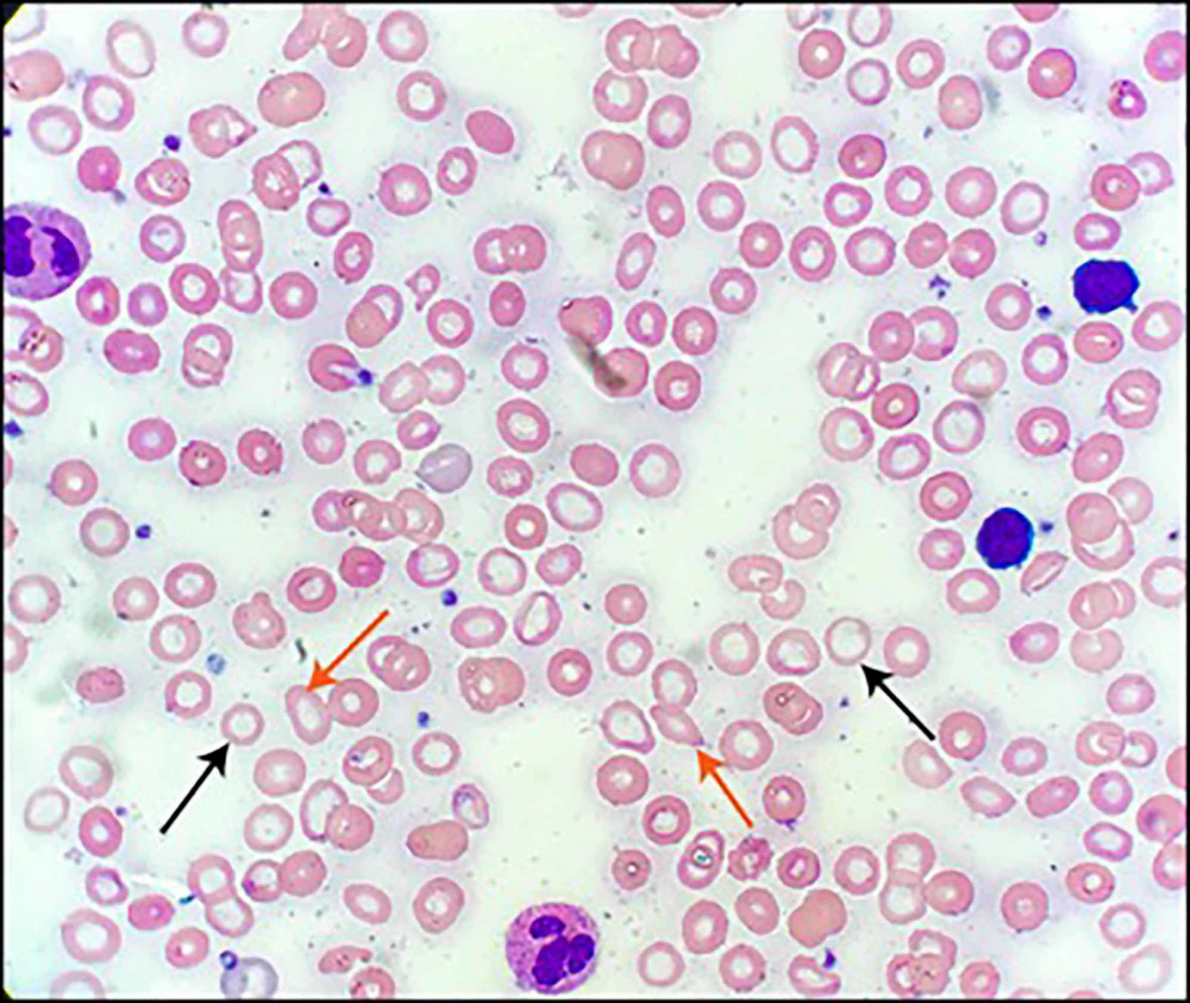
Peripheral blood smear of the patient upon presentation Evidence of marked hypochromia (black arrows) and microcytosis, as well as anisopoikolocytosis (red arrows)

Additional laboratory testing revealed white blood cells (WBCs) 4.5 x 109/L, platelet count 332 x 109/L, serum iron 6 µg/dL, total iron-binding capacity (TIBC) 394 µg/dL, TIBC% 2, reticulocyte count 1.41%, and normal lactate dehydrogenase (LDH), haptoglobin, creatinine, electrolytes, and liver function tests. The peripheral blood smear (Figure 1) was notable for microcytosis, with prominent erythrocytic central pallor. The patient was transfused two units of packed red blood cells with an incremental increase in his hemoglobin to 7.8 g/dL.

A nutritional analysis obtained via a detailed dietary history revealed inadequate daily caloric intake, as well as insufficient quantities of all macro- and micronutrients, including zinc (Table 1). Zinc deficiency was confirmed with a serum zinc level of 52 µg/dl (70-150 µg/dL). In addition to oral iron supplementation, he was started on a multivitamin with zinc and discharged to home. At follow-up 19 days after discharge, the patient described a significant improvement in his fatigue and weakness and a rapid restoration of his sense of taste, which had led to increased nutritional intake and a 2.3 kg weight gain.

**Table 1:** Estimation of the patient’s total daily intake of various macro- and micronutrients based on the acquisition of a detailed dietary history obtained by dietetic specialists Recommended daily value of each nutrient for males aged 51-70 listed in the right column

Nutrient	Daily Total	Reference Total (51-70 y/o male) [4]
Kcal	436 Kcal	2000 Kcal
Protein	18.4 g	40 g
Carbohydrate	65 g	210-303 g
Fat	12 g	41-73 g
Sodium	632 mg	1500 mg
Potassium	521 mg	3400 mg
Calcium	45 mg	1000 mg
Phosphorus	306 mg	700 mg
Iron	1.8 mg	8 mg
Zinc	1.3 mg	11 mg
Magnesium	32.7 mg	420 mg
Vitamin A	54 µg	900 µg
Vitamin C	15.6 mg	90 mg
Vitamin E	0.5 mg	15 mg
Vitamin B1 (thiamine)	0.27 mg	1.2 mg
Vitamin B2 (riboflavin)	0.20 mg	1.2 mg
Vitamin B6	0.39 mg	1.7 mg
Vitamin B12	0.46 µg	2.4 µg
Folate	45.11 µg	400 µg

## Discussion

Zinc is an essential trace mineral whose abundance among transition metal ions in all living organisms is second only to that of iron [5]. It functions as a cofactor or structural stabilizer for more than 300 enzymes throughout the body and directs a wide array of biochemical and cellular processes, including signal transduction, ribonucleic acid (RNA) transcription, and cellular growth, division, and differentiation [5-6]. Interestingly, patients with IDA have been shown to have significantly reduced serum levels of zinc while IDA is significantly more prevalent in patients known to have zinc deficiency [7-9]. More specifically, zinc deficiency has been speculated to contribute to the development of IDA through the inhibition of the intestinal absorption of iron as well as its mobilization from tissue stores [10].

Zinc deficiency can cause a variety of symptoms, including anorexia, lethargy, immune dysfunction, decreased wound healing, dermatitis, hypogonadism, and hypogeusia [11]. Our patient had weakness, fatigue, and weight loss, with a subnormal body mass index (BMI) and signs of cachexia on the physical exam. His long history of hypogeusia was a clear factor in the development of his malnutrition. Whether hypogeusia-induced malnutrition was the chief cause of his IDA is unclear. IDA is frequently seen in patients with negative upper and lower gastrointestinal (GI) endoscopic studies and is often attributed to small intestinal pathologies such as angiodysplasias, Crohn’s disease, celiac disease, and jejunal or ileal adenocarcinoma. Our patient did not undergo diagnostic testing aimed at assessing such pathologies. However, our nutritional analysis, with an estimated 1.8 mg daily iron intake (compared with the recommended daily intake of 8 mg), suggests that his IDA could have been entirely or largely a result of inadequate dietary iron. If dietary insufficiency was not the sole cause of his IDA, it was certainly a contributing factor.

Gustatory dysfunction manifests as a spectrum of taste disturbances, ranging from the relatively more common hypogeusia (a diminished sense of taste) and dysgeusia (altered perception of taste) to the more rare ageusia, a complete loss of taste [12]. In the USA, the prevalence of taste disturbance in the general population is 17.3% [13], but the vast majority of cases are caused by primary disturbances of olfaction [14]. Primary gustatory deficiencies are more frequently associated with malnutrition and diminished quality of life and can have a variety of etiologies, including poor oral hygiene or oral infection, sequelae of surgery or radiation, medication (e.g., chemotherapeutic drugs, angiotensin-converting enzyme (ACE) inhibitors, angiotensin II receptor blockers, macrolides, fluoroquinolones, and anticonvulsants, among others), trauma, renal failure, cancer, and zinc deficiency [15].

The molecular underpinnings of zinc deficiency-mediated hypogeusia are not fully understood, but a generous body of research has tied this phenomenon to various isoforms of human salivary gland-specific carbonic anhydrase, a zinc metalloenzyme whose expression and function have been shown to depend on ample concentrations of zinc [16-18]. It has been postulated that zinc supplementation may restore taste sensation in patients with both idiopathic and zinc deficiency-associated taste disturbances. Although the results of clinical trials have been inconsistent, a comprehensive Cochrane meta-analysis reveals some evidence to support the use of zinc supplementation to treat taste disturbances as measured by objective descriptors of taste acuity, particularly in zinc-deficient cohorts [3,19-20]. Our zinc-deficient patient’s chronic hypogeusia improved rapidly with zinc supplementation.

## Conclusions

Concurrent zinc deficiency should be considered in the evaluation of iron deficiency anemia, especially in patients with taste disturbances. Hypogeusia from zinc deficiency can lead to significant nutritional deficiencies and may cause or aggravate iron deficiency.
